# Structure–Property Linkage in Alloys Using Graph Neural Network and Explainable Artificial Intelligence

**DOI:** 10.3390/ma18163778

**Published:** 2025-08-12

**Authors:** Benjamin Rhoads, Abigail Hogue, Lars Kotthoff, Samrat Choudhury

**Affiliations:** 1 Department of Mechanical Engineering, University of Mississippi, University, MS 38677, USA; bmrhoads@go.olemiss.edu (B.R.);; 2 Department of Electrical Engineering and Computer Science, University of Wyoming, Laramie, WY 82071, USA

**Keywords:** graph neural network, deep learning, structure–property linkage, machine learning, nickel–aluminum, phase field simulation

## Abstract

Deep learning tools have recently shown significant potential for accelerating the prediction of microstructure–property linkage in materials. While deep neural networks like convolution neural networks (CNNs) can extract physics information from 3D microstructure images, they often require a large network architecture and substantial training time. In this research, we trained a graph neural network (GNN) using phase field generated microstructures of Ni-Al alloys to predict the evolution of mechanical properties. We found that a single GNN is capable of accurately predicting the strengthening of Ni-Al alloys with microstructures of varying sizes and dimensions, which cannot otherwise be done with a CNN. Additionally, GNN requires significantly less GPU utilization than CNN and offers more interpretable explanation of predictions using saliency analysis as features are manually defined in the graph. We also utilize explainable artificial intelligence tool Bayesian Inference to determine the coefficients in the power law equation that governs coarsening of precipitates. Overall, our work demonstrates the ability of the GNN to accurately and efficiently extract relevant information from material microstructures without having restrictions on microstructure size or dimension and offers an interpretable explanation.

## 1. Introduction

Determining the processing–microstructure–property (PMP) linkage plays a vital role in material design. In particular, the size, shape, and spatial distribution of microstructural features are known to affect material properties [1]. For example, in the case of metals and alloys, formation and subsequent growth of precipitates are known to affect the strength of a material. Phase field (PF) modeling based on fundamental principles of thermodynamics and kinetics has been successfully applied to provide insight into the evolution of precipitates in alloys [2] and link them to the mechanical strength of the alloys [3]. PF simulation provides microstructure evolution in alloys [4] by solving differential equations, but such calculations are often computationally expensive, particularly in 3D. More recently, it has been observed that machine learning (ML) tools, when trained on phase field simulated microstructures, can significantly accelerate the prediction of microstructure evolution at a fraction of the computational cost [5].

In this work, we focus on nickel–aluminum (Ni-Al) alloys, which are important high-temperature structural materials due to their low density, high strength, and excellent oxidation resistance [6]. Their microstructure consists of ordered L1_2_ Ni_3_Al (γ′) precipitates coherently embedded in a disordered FCC Ni-rich (γ) matrix [4]. The morphology, size, and distribution of these γ′ precipitates are the primary factors governing the strength, particularly under high-temperature loading. The microstructure–property relationships of these alloys can be determined using machine learning, which can play a critical role in optimizing alloy performance for aerospace and turbine applications.

There are two common approaches to predicting material properties with machine learning: The first is to use explicitly defined or extracted dataset features [7], like elemental properties and electronic and ionic attributes, to predict material properties like band gap energy. This typically involves ML tools like linear regression (LR), extra trees (ET) [8], and many others [9]. The second approach is to use high-dimensional data like 2D or 3D images, or continuous spectral measurements [10], which often require the use of deep learning (DL) tools like convolutional neural networks (CNNs). This approach has commonly been used to predict the material properties of simulated microstructures and experimental micrographs [11]. The primary difference between these two approaches is that the former feature-statistics-based method has simpler, but more interpretable, models, while deeper layers in DL models are much more complex and less interpretable. For example, a CNN, which is often thought of as a ‘black box,’ can achieve very high accuracy but has difficulty explaining its predictions [12]. In contrast, for feature statistics methods, while domain knowledge is required to know which features to extract, the model can be explained more intuitively in terms of those features. For example, feature importance studies can be performed with models like support vector machines (SVMs) and neural networks (NNs) [13]. Overall, choosing an approach depends on the complexity of the data and the accuracy desired; therefore, an ML model that can make predictions of high-dimensional data with high accuracy, as well as effectively explain such predictions, is necessary.

There is currently a significant effort to reduce the computational requirements of CNNs and expedite the training process [14,15]. Instead of using such high-dimensional networks like a CNN, there is a need for an alternate neural network that is less computationally expensive and does not require high-end GPUs. This would provide a pathway to the scientific community in the ML field to better process their data and develop high-fidelity models from it.

In this research, we demonstrate the utility of a graph neural network (GNN) as an alternate explainable approach that requires significantly reduced GPU resources in comparison to CNNs. GNNs process graphs, which are data structures made of individual nodes with different features, and edges representing the relationships between nodes [16]. GNNs have been successfully used in several material applications [17], such as coarse graining molecular systems to expedite molecular dynamics simulations [18]. GNNs have also been used to effectively predict the mechanical properties of polycrystalline materials where each grain is represented by a node and graph edges connecting nodes represent the grain boundaries [19]. Polycrystalline microstructures can also be converted into graphs and were previously used by a GNN to predict the magnetostriction of the material with about 10% error [20]. Additionally, integrated gradient analysis was used to determine the features in the microstructure that were most important in predicting magnetostriction. In this study, we used a GNN to predict the strengthening due to precipitate coarsening for phase field generated microstructures of Ni-Al alloys by representing high-dimensional microstructure as graphs with interpretable features.

In this work, we created a graph dataset from PF-generated microstructures of Ni-Al alloys by assigning a node to each precipitate in the microstructure and trained a GNN to predict the change in strength due to precipitate coarsening. We will demonstrate the equivalent/superior accuracy of the GNN compared to feature-statistics-based models and a high-dimensional-based CNN. Further, we will demonstrate superior generalizability with the GNN using train and test datasets comprising of microstructures of different sizes and dimensions. Later, we will also show the reduced computational resources required to train and test the GNN compared to the CNN.

Saliency analysis (SA), an explainable AI feature importance method, has been previously used in combination with CNNs to show the regions that have the most influence on the neural network prediction [21]. In this work, we have shown that when using SA in combination with a GNN, it is possible to find the importance of each node feature on predicted strengthening, which can help us understand the underlying physics governing material evolution. Unlike previously published research [22], in this work, we have also validated our results with a known equation for strengthening of alloys [23] and demonstrated that using feature importance with a GNN yields more interpretable results than when feature importance is used with a CNN.

Lastly, we used BI to determine the coefficients of the power law equation [24] that governs the growth of precipitates as a function of time. BI uses Bayes’ theory to estimate the coefficients of terms in an equation and their uncertainties [25] and has been previously used in parameter estimation for physics models [26]. Agreement between the BI-calculated coefficients with the known values of the coefficients confirms the accuracy of our PF data.

Overall, we demonstrate superior accuracy and generalizability while enabling unique insights with explainable AI using GNN. In addition, our proposed GNN-based approach uses fewer resources and can be run without access to expensive GPUs. This can make it easier to develop property prediction models of material microstructures with readily available CPUs.

## 2. Materials and Methods

In this work, we utilize a GNN with a PF-generated dataset of Ni-Al alloy microstructures converted into a graph dataset to predict the change in strength of the alloys from precipitate coarsening. Also, we use feature importance to find the most important features in the strengthening and BI to fit the precipitate growth to a power law equation. Finally, we compare the performance and speed of the GNN to other state-of-the-art feature-based ML tools and a comparable CNN. These methods are conducted to showcase the benefits of using GNN for materials microstructure over other ML tools.

### 2.1. Phase-Field Generation of Ni-Al Alloys

**Figure 1:**
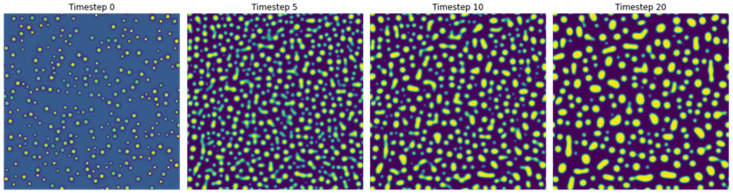
Example of microstructure evolution of Ni-Al during precipitate coarsening, simulated with the phase field methods described in Section 2.1.

Microstructures of Ni-Al were generated with a phase field model governed by Cahn–Hilliard kinetic Equations (1) and (2) [4,27,28]:

(1) $$\frac{\partial c}{\partial t}=\nabla \cdot \left(M\nabla \frac{\partial F}{\partial c}\right)=\nabla \cdot \left(M\nabla \frac{\partial f}{\partial c}\right)$$

(2) $$\frac{\partial \phi_{i}}{\partial t}=-L\frac{\partial F}{\partial \phi_{i}}=L\left(\varepsilon^{2}\nabla^{2}\phi_{i}-\frac{\partial f}{\partial \phi_{i}}\right)$$

where *c* is concentration of Al, ϕ is the order parameter, *F* is the total free energy, *f* is the free energy density, *t* is time, *M* is diffusion mobility, and *L* is a kinetic coefficient for order parameter relaxation. Further details regarding this model can be found elsewhere [4]. PF simulations were performed at a temperature of 1273 K and an Al concentration of 17.7%. Five image sequences with different random initializations were generated. Each sequence comprised of 21 images, with a time step of 0.8 s during coarsening of precipitates in Ni-Al alloys. Figure 1 shows microstructures at four different timesteps from one representative sequence obtained from phase field simulations.

### 2.2. Graph Construction

**Figure 2:**
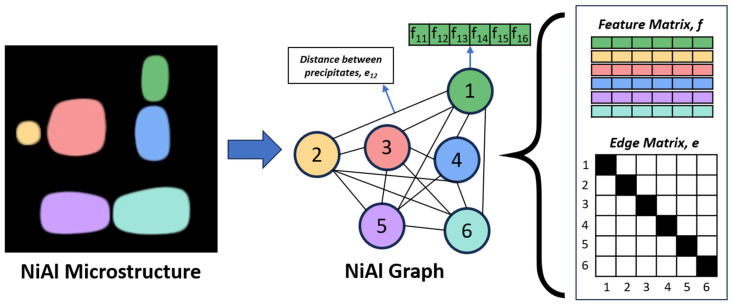
Conversion of Ni-Al microstructure to Ni-Al graph, including the node feature matrix, f, and the edge adjacency matrix, e.

A microstructure is converted into a graph by assigning each precipitate to a node. Each node has features that describe a precipitate, and this collection of node features is called the feature matrix, f. Additionally, interactions or relationships between nodes are represented by edges and are stored in the edge/adjacency matrix, e. In this study, we define 6 features for each precipitate: size, x, y, and z position of centroid, equivalent cube length, and extent. The size is equal to the area of precipitates for the 2D microstructure and the volume of precipitates for the 3D microstructure. The x, y, and z centroid positions are normalized by dividing them by the total number of pixels within the microstructure in that direction. The equivalent cube length is calculated by taking the square root of the size (for 2D microstructures) or the cubic root of the size (for 3D microstructures), and the extent is calculated by dividing the size either by the area or volume of the bounding box of the precipitate. We also define the edges between two nodes as the reciprocal of the distance between the centroid of the two corresponding precipitates. Only precipitates whose centroids are within 62 pixels of each other are given an edge value, which may include non-immediate neighbors. We tested distances of 42, 62, and 82 pixels and found no significant difference in the test mean absolute error (*MAE* = (1/*n*) ∗ Σ|*yᵢ* − *ŷᵢ|*, where *n* is the number of predictions, *yᵢ* is the true value, and *ŷᵢ* is the predicted value) of the GNN based on the distance used. Figure 2 shows a summary of the image-to-graph conversion. We used Python 3.8 with the StellarGraph 1.2.1 package for building the graphs and the GNN operations, and we used TensorFlow 2.8 for the rest of the operations.

### 2.3. Dataset Generation

A dataset of 2D and 3D Ni-Al microstructures of different sizes is created from PF simulations. Later, each microstructure is converted into a graph. We build 5 graph datasets (3 in 2D and 2 in 3D), each with 105 graphs constructed from five 21-microstructure sequences of Ni-Al alloys. Three graph datasets are made using 2D microstructures with sizes of 128 × 128, 256 × 256, and 512 × 512, and these microstructure datasets will be referred to in this paper as 2D-128, 2D-256, and 2D-512, respectively. The other two graph datasets are made using 3D microstructures with sizes of 64 × 64 × 64 and 128 × 128 × 128 and will be referred to in this paper as 3D-64 and 3D-128, respectively.

For each microstructure in these datasets, we calculate the strengthening of the alloy using Equation (3) [23]:

(3) $$∆\sigma_{mod}=0.0055M{\left(∆G\right)}^{3/2}{\left(\frac{f}{{0.5Gb}^{2}}\right)}^{1/2}b{\left(\frac{r}{b}\right)}^{\frac{3m}{2}-1}$$

where ∆G is the shear modulus difference between the matrix and precipitate (42.8 GPa), m = 0.85, M = 3.06, G is the shear modulus of Al (26.2 GPa), and b is the interatomic distance in slip direction of Al, which is 2.863 Å [23]. f is the area fraction, and r is the average cube length; both are calculated using a python script.

After calculating the strengthening of the alloy in the dataset, we sort them in order of increasing strengthening and then put every fifth microstructure in the test set while keeping the rest within the training set. We did this five times, each time offsetting the test data by one, resulting in five folds, each with an evenly distributed training set of 84 microstructures and a test set of 21 microstructures, allowing us to make consistent comparisons of model performances.

### 2.4. Graph Neural Network (GNN)

**Figure 3:**
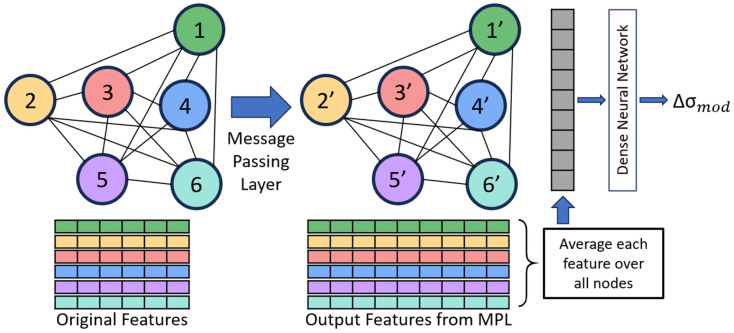
Overview of graph neural network, including message-passing layer, node feature averaging, and dense neural network.

We built a commonly used regression graph neural network that implements message-passing layers (MPLs), averages the features over all nodes, and then uses a dense neural network to predict the strengthening as shown in Figure 3 (additional description of GNN in Appendix A). In the MPL, information from features is passed between connected nodes and later aggregated, which allows information from neighboring nodes to be included in the updated features [29]. The next step returns the average value of each feature over all nodes, and the average feature values are used as input into the dense neural network. This contrasts with recent GNNs for predicting properties from microstructures, which concatenate the features of each node after the last MPL before being fed into the dense neural network [20], restricting all the graph datasets to have the same number of nodes. By taking the average feature values over all nodes, our GNN architecture makes it possible to train and test on graphs with any possible number of nodes. This means the graphs in the dataset can be constructed from microstructures of any size or dimension and can predict the properties of alloys with other microstructure sizes and/or dimensions, thus making this GNN significantly more generalizable than previous models.

#### GNN Parameters and Architecture Optimization

In this study, we investigated ten GNN architectures with varying complexity to determine the most appropriate model for our dataset. The size of each MPL and dense neural network layer in these GNNs is listed in Table 1. In this study, we use a dropout of 0.2 in each MPL and ReLU activation in each MPL and dense layer. Further, a learning rate of 0.001 with the Adam optimizer was used. We observed that the learning rate and dropout had little impact on GNN performance. ReLU activation function was used for its computational efficiency. We initialized the model weights with random Xavier distributions and used the MAE loss function between the true and predicted strengthening. Each model in Table 1 is trained until the test MAE does not improve for 150 epochs and is relatively close to the train MAE to ensure there is no overfitting. Using the 2D-256 dataset with the 80–20 train–test split described in Section 2.3, we repeat 5-fold cross validation 5 times for a total of 25 comparisons for all GNN architectures. We determined the ranks of each GNN’s minimum test MAE over all 25 tests and used the architecture that achieves the best average rank for the remainder of the study.

### 2.5. Feature Importance

Using the optimized graph construction method and the GNN, we perform feature importance to determine which of the 6 manually defined features (size, x, y, and z position of centroid, equivalent cube length, and extent) are most important for predicting strengthening. The feature importance was performed by taking the gradient of the GNN strengthening prediction with respect to each feature in each node to obtain the importance score of each feature at each node. Then, we added all the individual feature importance scores for each feature across all nodes to determine a cumulative importance score of each feature. By comparing the cumulative score of each feature to the variables present in commonly used strengthening equation (Equation (3)), it is possible to determine if the GNN can extract important features linked to strengthening.

### 2.6. GNN Performance

To demonstrate the robustness of the GNN, we analyze the ability of the GNN to make predictions on graph datasets that are created from microstructures of different dimensions and sizes. We train and test our GNN on the 2D-128, 2D-256, 2D-512, 3D-64, and 3D-128 datasets, using the train–test approach presented in Section 2.3, and report the average test MAE of all 25 runs. We also investigate cases where we train on one or more dataset and test on separate dataset(s), and we measure the average test MAE. In these cases, there is only one train–test fold, but we still train and test on this fold with 5 different random weight initializations and take the average MAE from the 5 runs. These results will demonstrate the accuracy in predicting the strengthening when the training is performed on one dataset, while the testing is conducted on a different graph dataset comprising of microstructures of different dimensions and/or sizes.

### 2.7. Comparison of GNN to Other Machine Learning Models

Later, we compared the performance of our GNN to other state-of-the-art ML tools to identify the types of applications for which GNNs are the most optimal ML tool choice. To determine the performance of the state-of-the-art ML tools, we first extract the average equivalent cube length, area fraction, and average extent from our 2D-256 and 3D-128 datasets and predict the strengthening using AutoGluon 1.4 [30]. AutoGluon is a tool for training a variety of ML models on tabular data with minimal python code. In this study, we recorded the model used by Autogluon with the lowest test MAE as the baseline. We also trained a 2D and 3D CNN to predict strengthening with the images from 2D-256 and 3D-128 datasets, respectively. Each CNN uses 3 × 3 convolution kernels with stride 1 and ReLU activation, followed by max pooling layers of size 2. After the final convolution layer, the output is flattened and passed through dense layers. All models were trained until the MAE failed to improve for 150 consecutive epochs. We increased the complexity of the 3D CNN architecture until the memory of our Nvidia Tesla A100 40GB GPU was completely utilized during model training and/or testing. This ensures our GNN and CNN models have similar memory requirements. We determine the optimum CNN architecture based on the lowest error listed in Table 2. All MAEs reported are the average minimum MAE of 25 total runs obtained by repeating 5-fold cross-validation 5 times.

Unlike CNNs where dimensionality is related to the number of pixels, the number of node features governs the dimensions of a GNN. Hence, in the case of GNN, we significantly reduce the dimensionality of the data when we construct graphs from images. Compared to GNN, the complexity of the data used with AutoGluon is further reduced as we only utilize extracted average equivalent cube length, area fraction, and average extent. Therefore, the comparison of the various models used in this study can also provide insight regarding how the dimensionality of the data can influence the error in the predictive models utilized.

### 2.8. Bayesian Inference for Power Law Equation

The final component of this study will be to extract the coefficients of the power law equation governing precipitate growth during coarsening in alloys [24]

(4) $$r_{t}={\left(r_{0}^{n}-kt\right)}^{\frac{1}{n}}$$

where $r_{t}$ is the equivalent cube length at time t, $r_{0}$ is the equivalent cube length when coarsening starts, k is the coarsening rate constant, and *n*~3. We utilize BI, which uses Bayes’ theorem to update prior distributions for given coefficients to find the most likely values of coefficients [31]. Using BI, we determine the most likely values of n and k for a microstructure dataset generated from our phase field simulations. For this data, we use Ni-Al microstructures generated with the same phase field parameters as before but extend the real simulation time to 120 s and record the average equivalent cube length every 0.4 s.

## 3. Results

In this study, we use feature importance to determine the most important node features that governs strengthening. Later, we optimize the architecture of the GNN and compare this GNN to state-of-the-art ML tools and CNNs. Additionally, we use BI to determine the coefficients of the power law equation governing the precipitate growth.

### 3.1. Node Feature Importance

**Figure 4:**
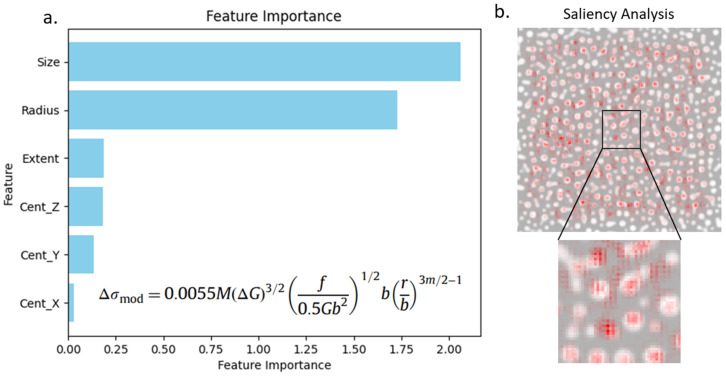
Comparison of feature importance capabilities of (**a**) GNN and (**b**) CNN. (**a**) Importance of each node feature to predict strengthening. Equation used to calculate strengthening is also shown; (**b**) importance of each pixel in the microstructure obtained from CNN-based feature importance.

The importance of each of the six node features towards strengthening is shown in Figure 4a. This approach was used to gain physical understanding of the main features involved in strengthening. It can be observed that the two features with the highest importance obtained from our explainable AI tool with the GNN were the size and radius, which are also the two independent variables in the strengthening equation. Similarly, the x, y, and z position of the centroid and the extent are not in the strengthening equation, which agrees with the low importance values obtained from feature importance.

Figure 4b shows the feature importance analysis with an optimized CNN. The red spots represent pixels that have the most importance in predicting strengthening. Although some precipitates and part of the matrix are considered important, there is no recognizable pattern. Unlike the GNN, CNN-based feature importance cannot provide humanly interpretable features in its explanation.

Feature importance was also used to determine the importance of node edges (interactions between precipitates) and nodes (individual precipitates). In both cases, there was no clear trend in the observed importance values, which also agreed with our current understanding that individual precipitates as well as precipitate–precipitate interactions have a negligible influence on strengthening.

### 3.2. GNN Optimization

**Figure 5:**
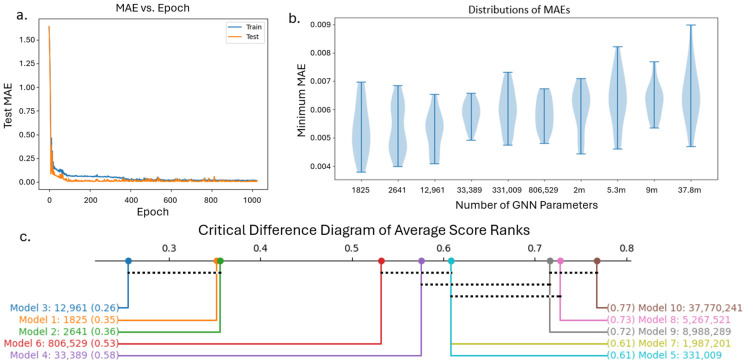
(**a**) Example train–test loss curve for a GNN; (**b**) distribution of minimum test MAEs from 25 runs of each GNN model; (**c**) critical difference plot showing average rank scores, with the black dotted lines connecting models within one critical distance, indicating those models are not significantly different.

To ensure that our GNN model is not overfitting, we compared a representative loss curve during training and testing of a GNN model (Figure 5a). Next, we optimized the architecture of the GNN based on the minimum test error in its predictions. We tested the performance of different GNN architectures by changing the number of units in the graph convolution and dense layers, as listed in Table 1. We repeat this 5-fold cross validation 5 times for each of these ten GNN architectures and show the distribution of the minimum test MAE over all 25 runs (Figure 5b). It can be observed that models 1, 2, and 3 have lower average MAEs compared to the remaining seven models.

The performance of these ten models was further analyzed based on average rank. The average rank is calculated by taking the average of ranks based on MAE over all 25 runs for each model as shown in Figure 5c. The horizontal dotted black lines connect the models that have average ranks within one critical difference of each other with 95% confidence. The number of parameters for each model is also presented. This plot shows that models 1, 2, and 3 performed better than all other models, but it is not possible to conclude any significant difference between the test MAE of model 1, 2, or 3. However, as model 3 had the lowest average rank, we continue the rest of this study using the architecture of GNN model 3, which has 12,961 trainable parameters.

### 3.3. GNN Performance with Different Sizes and Dimensions of Microstructure Datasets

**Figure 6:**
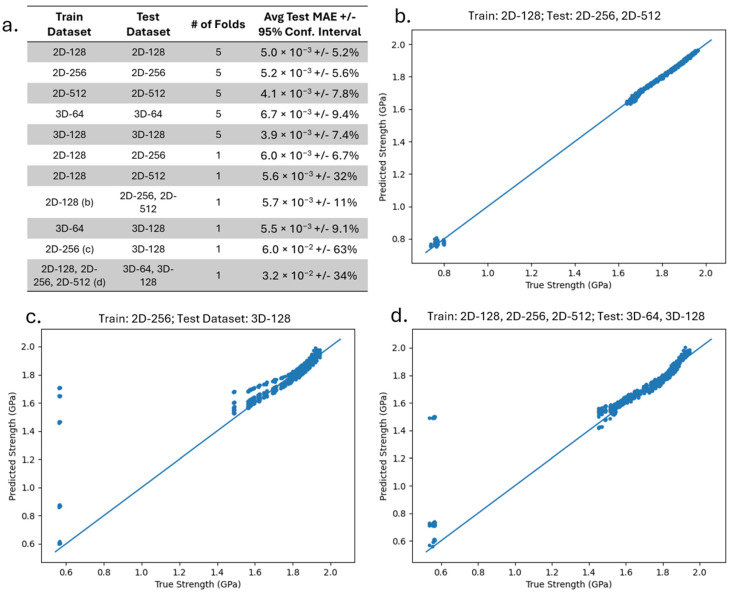
(**a**) Average test MAE and 95% confidence intervals for different train/test dataset configurations. (**b**–**d**) Predicted vs. true strengthening plots for selected datasets. The dataset(s) used to train and test the GNN are also presented.

We then tested the performance of the optimized GNN on multiple 2D and 3D microstructure datasets. In Figure 6a, we list the datasets used during training and testing, the number of folds used, and the average test MAE with the 95% confidence interval. All folds are repeated five times with different random Xavier distribution weight initializations. It can be observed from Figure 6a that the test MAE and the confidence interval increase when the train and test graph datasets are constructed from microstructures with a different size or dimension.

Figure 6b–d show the true strength (calculated using Equation (3)) vs. GNN predicted strength of the test data for three representative dataset(s) listed in Figure 6a. Figure 6b shows the true–predicted plot of the test graphs, which were constructed from microstructure datasets of size 2D-256 and 2D-512, after training the GNN on graphs constructed from 2D-128 microstructures. It is interesting to observe that the average test MAE for 2D-256 and 2D-512 is similar and is indifferent to whether it is trained on 2D-128, 2D-256, or 2D-512. It can be concluded that the GNN can successfully be trained on graphs based on microstructures of one set of sizes and can be used effectively to predict properties of alloys with larger size microstructures without affecting model performance.

Figure 6c,d show the results when the train and the test dataset are of different dimensions. In Figure 6c, the training data were constructed from 2D-256 microstructures, while the test data was constructed from 3D-128 microstructures. In Figure 6d, all 2D datasets (2D-128, 2D-256, and 2D-512) and 3D datasets (3D-64 and 3D-128) were used in the train and test dataset, respectively. Unlike the case when the training and test datasets are of different sizes, an increase in MAE and variance is observed when the train and the test dataset are of different dimensions. One possible source for the increase in error in the latter case is that the size feature is represented by the area and volume for a 2D and 3D microstructure, respectively. Further, the 3D morphology of the precipitates is not well represented within the 2D microstructure training datasets.

It is also worth noting that in Figure 6c,d, there are significant errors in some of the predictions for smaller values of true strength, which is likely because there was a lot less data available at these smaller strengthening values. This can also occur when training is stopped early once the test loss has not improved after 150 epochs for a certain set of random weight initialization (see Appendix A). Increasing patience is one possible way to mitigate this problem. However, increasing patience would also increase the training time for all models. One could also train the model until the MAE falls below a certain threshold, but this threshold may be difficult to determine. For simplicity, we did not change the patience for any of our tests to show an accurate comparison of all models. Nevertheless, these results demonstrate an advantage to the GNN over the CNN in its ability to be trained and tested on graphs processed from multiple dimensions and sizes and still make accurate predictions.

### 3.4. Comparison of GNN with ML Tools and CNN

**Figure 7:**
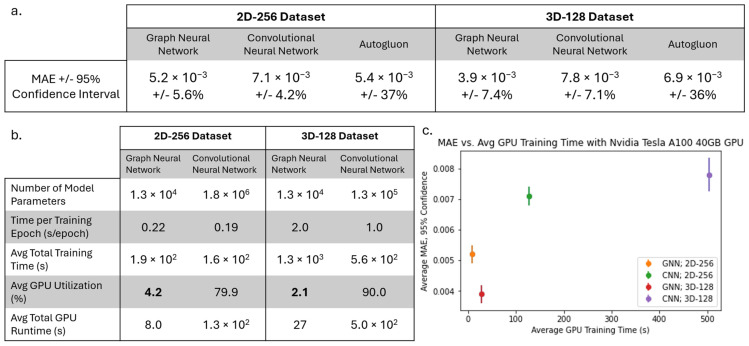
(**a**) Average MAE and 95% confidence intervals for GNN, CNN, and AutoGluon on 2D and 3D datasets. (**b**) Model size, training time, and GPU usage for GNN and CNN. Minimum GPU utilization is highlighted in bold for each dataset. (**c**) MAE vs. GPU training time for GNN and CNN on 2D and 3D datasets.

To study the prediction capabilities of the GNN in the context of other ML tools, we compared the performance of the GNN to a CNN and other commonly used ML models like XGBoost, ET, and KNN using AutoGluon. We tested our GNN and CNN on the 2D-256 and 3D-128 dataset, repeating 5-fold cross-validation 5 times, and recorded the average test MAE of all 25 runs in Figure 7a. Later, we used AutoGluon on the tabular data with 5-fold cross validation, but with no repetitions, and presented the average MAE and confidence interval in Figure 7a. We see that the GNN achieves a better test MAE than the CNN, and a test MAE similar to the best model from Autogluon, but with a much lower confidence interval. This shows the ability of the GNN to make predictions with both higher accuracy and higher confidence compared to other commonly used ML tools.

**Figure A2:**
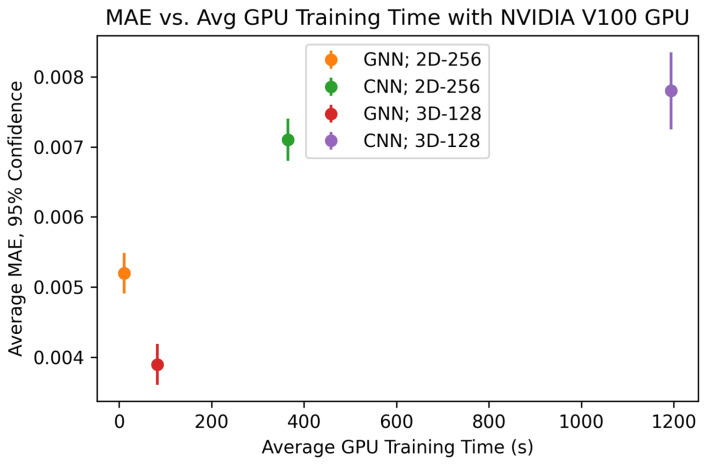
Average MAE over 5-fold cross validation repeated 5 times (25 total runs) with 95% confidence interval vs. average GPU training time of CNN and GNN on 2D and 3D datasets, trained using one NVIDIA V100 GPU.

**Figure A3:**
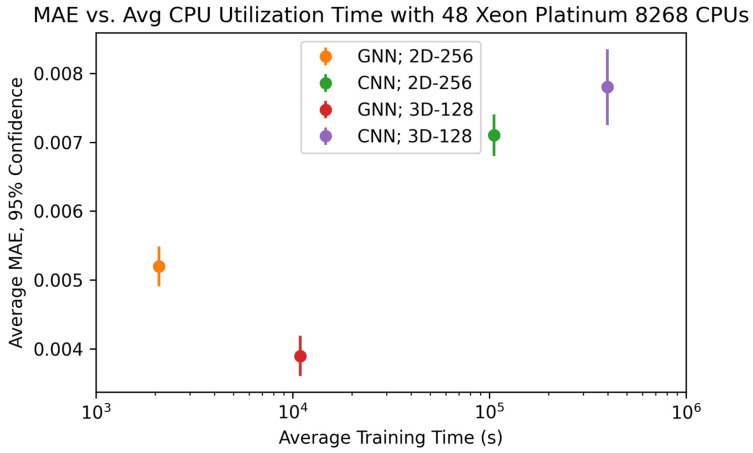
Average MAE over 5-fold cross validation repeated 5 times (25 total runs) with 95% confidence interval vs. average GPU training time of CNN and GNN on 2D and 3D datasets, trained using 48 Xeon Platinum 8268 CPUs.

Additionally, for the GNN and CNN, we present in Figure 7b the size of each model (the number of model parameters), the average seconds per epoch during training, the average total train time, the average GPU utilization, and the average total GPU train time (total train time multiplied by GPU utilization). We observed that the GNN model has fewer trainable parameters than the 2D CNN and 3D CNN. While the GNN takes more overall time to train, it requires significantly less computational time on a GPU than CNNs. Overall, we found that the GNN operations do not efficiently utilize GPUs like CNNs, which are very well suited for GPUs [32]. To further demonstrate the utility of GNN, we plotted the MAE vs. GPU train time in Figure 7c for the GNN and CNN on the two different datasets, where the GPU for both is an NVIDIA A100 40 GB GPU. In this figure, the best models are GNN based as shown at the bottom left of the figure. The performance of the GNN and CNN was compared on CPUs as well as on a GPU with different architecture. For example, we measured the GPU utilization time using an NVIDIA V100 GPU as well as the computational time using 48 Xeon Platinum 8268 CPUs, and the results are presented in Figure A2 and Figure A3 in Appendix B. We found that when run on CPUs, the GNN trains about 30 times faster than the CNN. This is because the graph dataset and GNN are much smaller compared to the image dataset and CNN. We are not aware of any efforts to more efficiently train GNNs with GPUs; therefore, this may be a potential area of later research. In any case, it can be concluded that the GNN can be trained much faster than a CNN for our microstructure datasets on readily available CPUs, without requiring more expensive GPUs.

### 3.5. Determining Power Law Coefficients Using Bayesian Inference

**Figure 8:**
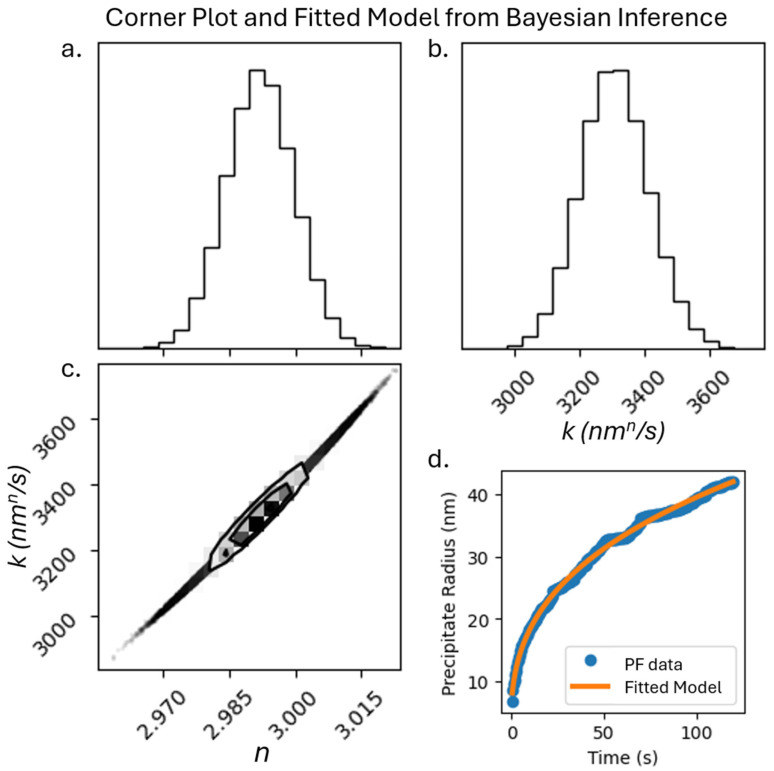
Corner plot of Bayesian Inference results. (**a**) Range of possible values for *n*; (**b**) range of possible values for *k* and (**c**) range of possible values for both *n* and *k*; (**d**) plot of power law model with predicted coefficients compared to average precipitate size from phase field (PF) simulations.

The primary emphasis of recent ML tools is focused on achieving a high predictive accuracy of property from microstructures and/or chemistry with limited focus on discovering new underlying materials physics. In this section, we use ML tools to develop an understanding about the principles governing microstructure evolution. As a first step, we used explainable artificial intelligence tools such as BI to extract parameters in a commonly known power law equation that governs precipitate evolution at higher temperatures. We used the evolution of average precipitate size within the 2D-256 microstructures to determine the coefficients *n* and *k* in Equation (4) using BI. We estimate the most likely values of *n* and *k* to be 2.992 and 3300 nm*^n^*/s, respectively. Figure 8a and Figure 8b show the distribution of possible values for *n* and *k*, respectively. The distribution of possible values for *n* and *k* is a narrow distribution, showing a high correlation between the two parameters (Figure 8c). Our results confirm the typically known value of *n*~3 in the power law equation. Later, we used these ML-predicted values of *n* and *k* to predict the size of the precipitates as a function of time and compared our results with the average size of the precipitates obtained from phase-field simulations (Figure 8d).

## 4. Discussion

The results from this study have several promising implications in materials science. While recent studies apply GNNs to inherently graph-structured data such as atomic or molecular systems [18,33], our approach demonstrates that high-dimensional image-based microstructures can be effectively converted into graphs and that GNNs can still achieve high prediction accuracy on these derived graph representations. Additionally, we show the importance of node features to predict strengthening, which was confirmed by parameters in a typically used strengthening equation. Feature importance was also applied to find the importance of the individual precipitates and the edges between them. While no clear trend is observed for the case of individual precipitates and the edges between them, it is possible to use the same tool with other microstructure graph datasets to determine the features, nodes, and interactions between nodes that govern the prediction of material properties. For example, in grain growth [34], the evolution of grains depends on nearby features, like neighboring grains or different types of grain boundaries. Other machine learning tools including physics-regularized interpretable machine learning microstructure evolution (PRIMME) [35], as well as using feature importance with a CNN, can provide heat maps showing the importance of pixels within microstructures. However, using feature importance with a GNN can provide more specific importance scores of microstructure components (graph nodes), their features, and the interactions between components (graph edges). Another promising method is to embed physics components in the graph structure, like energy components and stress fields, which may help provide more scientific insights using explainable artificial intelligence methods. Therefore, usage of feature importance with GNNs has potential to increase our understanding of the underlying physics principles governing the properties and evolution of microstructures.

Another observation we made in this study was the difference in computational resources during training, which is a function of the complexity of the GNN compared to the CNN as well as the efficiency of each model on a GPU. Because the Ni-Al microstructures had very simple precipitates, there was a relatively small amount of data needed to fully describe each microstructure with a graph. Because of this, the optimized GNN architecture only had 12,961 trainable parameters, a fraction of the parameters needed in the 2D and 3D CNNs. However, for a more complicated microstructure with several microstructural features (grains, precipitates, domains, etc.), we expect that both the complexity of the graph and the GNN will increase. This may lead the computational requirements of the GNN to approach and possibly surpass that of the image data and CNN. Conversely, for even simpler microstructures compared to the microstructures presented in the work, we expect the graph to be less complex. This idea can be further studied to understand the degree to which GNN and graph complexity is dependent on microstructure complexity and can be a major factor in determining whether GNN is the best option for a given microstructure-based property prediction.

The final benefit of using the GNN with microstructure data is the ability to extrapolate the dimensions and sizes of the microstructures used to construct the graph dataset. This shows that a single GNN model can learn the necessary physics for predicting the strengthening, regardless of the size or dimension of the microstructure the graph is trained on. This suggests that GNNs can be a more efficient and generalizable material characterization tool or surrogate model for physics simulations, whereas other deep-learning-based models are restricted to predictions that have only a single dimension and size [5]. Therefore, while it can take days to months to generate large 3D microstructures with PF, and then additional days to train a DL model on the data, a GNN can be trained in much less time on data generated in a fraction of the time (e.g., smaller size in 2D) and can make predictions for any other dimension or size. Additionally, the phase field generated microstructures used in this study were previously validated with TEM dark-field electron micrographs at various times during evolution by comparing the same morphological patterns in both simulation and experiment [4,24]. Based on this validation of the data used in this study, we expect a similar GNN can be trained on experimental micrographs if the dataset is of similar quantity and quality. These benefits of GNN can significantly expedite the material design and optimization process, allowing for rapid exploration of the microstructure–property relationship in materials.

## 5. Conclusions

We constructed graph datasets from microstructures of phase field-generated precipitate coarsening in Ni-Al alloys and trained a GNN on the graphs to predict strengthening during microstructure evolution. We then used feature importance to accurately determine which precipitate features govern the prediction of strengthening. Later, multiple GNN architectures were tested to determine which model complexity made the most accurate strengthening predictions. We trained and tested the optimal GNN architecture on graph datasets constructed from 2D and 3D microstructures with multiple sizes and achieved a very low prediction MAE. We also observed that the GNN can be tested on graphs from microstructures of entirely different sizes and dimensions than the microstructures used to make the training graphs, showing that the GNN is inherently more generalizable than a CNN. In addition, the performance of the GNN is similar to that of state-of-the-art ML tools and a CNN, and we find that the GNN requires significantly less GPU and CPU utilization than the CNN, providing the same or better prediction accuracy at a fraction of the computational cost. Finally, we use Bayesian inference to confirm coefficients in the commonly used equation governing the size of precipitates during coarsening. Overall, we have clearly shown in this work that converting microstructure images into graphs for use in a GNN allows for accurate feature importance analysis, lower computational requirements, and notably more generalizability.

## Figures and Tables

**Table 1:** Size of message-passing layers and dense neural network layers in 10 GNN architectures and the corresponding number of trainable parameters.

GNN Model Number	Message Passing Layer Sizes	Dense Neural Network Layer Sizes	Number of Trainable Parameters in Model
1	[32, 32]	[16, 1]	1825
2	[32, 32, 32]	[8, 4, 1]	2641
3	[128, 64, 32]	[32, 16, 8, 1]	12,961
4	[128, 256, 512]	[256, 128, 32, 1]	33,389
5	[256, 256, 256]	[256, 256, 256, 1]	331,009
6	[256, 512, 512]	[512, 256, 64, 1]	806,529
7	[256, 512, 1024]	[1024, 256, 64, 1]	1,987,201
8	[512, 1024, 2048]	[1024, 512, 32, 1]	5,267,521
9	[512, 1024, 2048]	[2048, 1024, 64, 1]	8,988,289
10	[1024, 2048, 4096]	[4096, 2048, 1024, 1]	37,770,241

**Table 2:** CNN architecture optimization for 2D-256 and 3D-128 datasets. Each row shows the convolutional and dense layer configurations and the average minimum MAE over 25 runs. All models use ReLU activation, 3 × 3 kernels, stride 1, and max pooling of size 2. The error for the optimum CNN architectures are highlighted in bold.

2D-256 Dataset			3D-128 Dataset		
Number of Filters in Each 2D Convolution Layer	Dense Layer Sizes	MAE	Number of Filters in Each 3D Convolution Layer	Dense Layer Sizes	MAE
[16, 16, 16, 16]	[1]	1.1 × 10^−2^	[32, 32, 32, 32]	[1]	1.2 × 10^−2^
[32, 32, 32, 32]	[1]	1.0 × 10^−2^	[32, 32, 64, 64]	[1]	1.6 × 10^−2^
[64, 64, 64, 64]	[1]	9.1 × 10^−3^	[32, 32, 32, 32]	[64, 1]	1.5 × 10^−2^
[128, 128, 128, 128]	[1]	8.7 × 10^−3^	[32, 32, 32, 32]	[64, 32, 1]	1.0 × 10^−2^
[128, 128, 128, 128]	[128, 1]	1.0 × 10^−2^	[32, 32, 32, 32]	[32, 1]	1.1 × 10^−2^
[256, 256, 256, 256]	[1]	**7.1 × 10^−3^**	[48, 48, 48, 48]	[1]	1.1 × 10^−2^
[256, 256, 256, 256]	[256, 1]	8.1 × 10^−3^	[48, 48, 48, 48]	[64, 32, 1]	1.3 × 10^−2^
[512, 512, 512, 512]	[1]	1.3 × 10^−2^	[32, 32, 32, 32, 32]	[1]	1.6 × 10^−2^
[32, 32, 32, 32, 32]	[1]	1.2 × 10^−2^	[32, 32, 32, 32, 32]	[32, 1]	1.5 × 10^−2^
[64, 64, 64, 64, 64]	[1]	1.1 × 10^−2^	[32, 32, 32, 32, 32]	[64, 1]	1.1 × 10^−2^
[128, 128, 128, 128, 128]	[1]	8.1 × 10^−3^	[32, 32, 32, 32, 32]	[64, 32, 1]	**8.9 × 10^−3^**
[128, 128, 128, 128, 128]	[128, 1]	8.8 × 10^−3^	[48, 48, 48, 48, 48]	[1]	1.4 × 10^−2^
[256, 256, 256, 256, 256]	[1]	1.3 × 10^−2^	[48, 48, 48, 48, 48]	[64, 32]	1.5 × 10^−2^
[256, 256, 256, 256, 256]	[256, 1]	1.1 × 10^−2^			
[512, 512, 512, 512, 512]	[1]	1.6 × 10^−2^			
[128, 128, 256, 256, 512]	[256, 1]	1.5 × 10^−2^			
[64, 64, 64, 64, 64, 64]	[1]	1.1 × 10^−2^			
[128, 128, 128, 128, 128, 128]	[1]	1.0 × 10^−2^			
[128, 128, 128, 128, 128, 128]	[128, 1]	9.6 × 10^−3^			
[256, 256, 256, 256, 256, 256]	[1]	1.0 × 10^−2^			
[512, 512, 512, 512, 512, 512]	[1]	1.5 × 10^−2^			
[128, 128, 256, 256, 512, 512]	[256, 1]	9.2 × 10^−3^			

**Figure A1:**
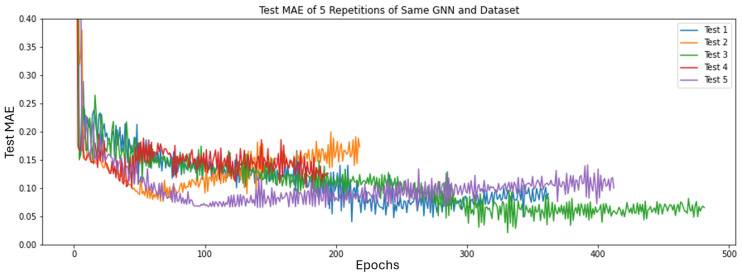
Test MAE while training GNN on 2D-256 dataset and testing on the 3D-128 data with 5 different random initial weight distributions. For the fourth initial weight distribution (Test 4), the test loss requires more epochs to decrease to an optimal loss than the patience specified (150), so the model stops training when the test MAE is relatively high.

## Data Availability

The original contributions presented in this study are included in the article. Further inquiries can be directed to the corresponding author.

## Appendix A. GNN Details

Using StellarGraph, a GNN is built with the GCNSupervisedGraphClassification message-passing-layers (MPLs) class and dense feed-forward layers. The GNN consists of either two or three message-passing layers (MPLs), and the average value of each output feature is taken across all nodes in the graph. The vector of average feature values is then used as input for the dense neural network comprising of two to four dense layers, where the final layer has one unit. All MPLs and dense layers use the ReLU activation function, and all MPLs use a dropout of 0.2. Adam optimizer is used with a learning rate of 0.001, and the MAE is used as the train and test metric. A patience of 150 is used, meaning the model is trained until the test loss does not improve for 150 consecutive epochs. The PaddedGraphGenerator class is used to batch the graphs, which all have different sizes, into batches of 30 graphs. In each epoch, random batches are taken from the training dataset without replacement until all training data has been used for training, and the model is then evaluated on the test data after each epoch. In Figure A1, we show the five MSE vs. Epoch curves associated with the results in Figure 6c in the main text and observe that Test 4 (in red) was stopped too early, resulting in poor performance.

## Appendix B. Additional Computer Usage Analysis

Figure A2 and Figure A3 plot the average test MAE over 25 total runs with a 95% confidence interval vs. the average GPU and CPU training time, respectively. Both figures show the same trend as the one provided in Figure 7c, where the GNN outperforms the CNN in test MAE as well as training time.
